# 30-Day Postoperative Complications After Surgical Treatment of Proximal Humerus Fractures: Reverse Total Shoulder Arthroplasty Versus Hemiarthroplasty

**DOI:** 10.5435/JAAOSGlobal-D-22-00174

**Published:** 2023-03-03

**Authors:** Michael Khazzam, Junho Ahn, Brian Sager, Stephen Gates, Megan Sorich, Nathan Boes

**Affiliations:** From the Department of Orthopaedic Surgery, Shoulder Service, University of Texas Southwestern Medical Center, Dallas, TX.

## Abstract

The purpose of this study was to evaluate risk factors associated with complications after reverse total shoulder arthroplasty (TSA) and hemiarthroplasty for the treatment of proximal humerus fractures.

**Methods:**

A retrospective review of the American College of Surgeons National Surgical Quality Improvement Program database was conducted. Current Procedural Terminology codes were used to identify patients treated for proximal humerus fracture with reverse TSA or hemiarthroplasty between 2005 and 2018.

**Results:**

One thousand five hundred sixty-three shoulder arthroplasties were conducted: 436 hemiarthroplasties and 1,127 reverse TSA. The overall complication rate was 15.4% (15.7% reverse TSA; 14.7% hemiarthroplasty) (*P* = 0.636). Most frequent complications included transfusion 11.1%, unplanned readmission 3.8%, and revision surgery 2.1%. A 1.1% incidence of thromboembolic events was noted. Complications occurred most frequently in patients older than 65 years; male; and patients with anemia, American Society of Anesthesiologists classification III-IV, inpatient procedure, bleeding disorders, duration of surgery >106 minutes, and length of stay >2.5 days. Patients with body mass index >36 kg/m^2^ had a decreased risk of 30-day postoperative complications.

**Discussion:**

There was a 15.4% complication rate in the early postoperative period. In addition, no notable difference was found in complication rates between groups (hemiarthroplasty: 14.7%; reverse TSA 15.7%). Future studies are needed to determine whether there is a difference between these groups in the long-term outcome and survivorship of these implants.

Proximal humerus fractures account for approximately 5% of all fractures and are the third most common fracture type in those older than 65 years.^1,2^ Management options are influenced by a number of factors, among which are fracture type and severity, patient age and comorbidities, patient's functional status, and surgeon experience.^3,4^ A number of treatment options exist, ranging from short-term immobilization with early range of motion and physical therapy to surgical management.^1^ Most proximal humerus fractures in the elderly population can be managed nonsurgically and with good functional outcomes.^3,5^

Given the difficulty in closed management of displaced three-part and four-part proximal humerus fractures, as well as the associated high risk of osteonecrosis, shoulder hemiarthroplasty has long been used for complex fracture types.^6^ Along with the advent of reverse total shoulder arthroplasty, both hemiarthroplasty (HA) and reverse total shoulder arthroplasty (RSA) have become surgical treatment options for proximal humerus fractures in the elderly patient population.

Recent epidemiologic data suggest that the use of RSA has become more prevalent, up by 406% over an 8-year span from 2005 to 2012 in the Medicare population, compared with a 47% decreased use of HA over the same period.^7^ Both patient factors and surgeon preference have been cited for the shift in implant utility.^8,9^

With good clinical outcomes, improved shoulder rotation, and relatively shorter surgical times, HA remains a viable option in the surgical management of complex, comminuted proximal humerus fractures.^10,11^ Outcomes after HA, however, correlate closely with anatomic healing of the tuberosities, with malposition leading to markedly worse functional outcomes and decreased shoulder range of motion.^3,10-12^ Because anatomic tuberosity healing is essential for restoration of rotator cuff function, the use of RSA for complex proximal humerus fractures can arguably mitigate the necessity of relying on anatomic tuberosity fixation.^3,13^ This factor, along with predictably good early and midterm clinical outcomes, makes RSA a particularly useful surgical option in elderly patients with complex proximal humerus fractures, particularly those with osteopenic bone.^14,15^ The purpose of this study was to evaluate the 30-day postoperative complication rate and associated risk factors in the early postoperative period after the surgical treatment of proximal humerus fractures with reverse total shoulder arthroplasty compared with hemiarthroplasty.

## Methods

This is a retrospective cohort study using the American College of Surgeons National Surgical Quality Improvement Program (ACS-NSQIP) database, a surgical registry that collects data from more than 600 hospitals in the United States, to identify patients who underwent either hemiarthroplasty or total shoulder arthroplasty for the treatment of a proximal humerus fracture between 2005 and 2018. Compared with healthcare databases that are claims-based, such as the Medicare database, the ACS-NSQIP has reviewers at each hospital site to collect patient information from randomly assigned patient medical charts 30 days before operation up to 30 days after the operation, regardless of the discharge date. Patient data are continuously collected and uploaded to a Health Insurance Portability and Accountability–compliant web-based registry. To improve comparability between the various participant sites, the cases included in ACS-NSQIP are case-mix–adjusted and risk-adjusted. The database includes more than 150 patient variables. However, variables that were not part of the inclusion/exclusion criteria, not pertinent to the goal of this study, or those that had missing values were not included in the analysis.

### Patient Selection Criteria

Patients who underwent surgery for the treatment of a proximal humerus fracture with either a hemiarthroplasty or total shoulder arthroplasty were extracted using Current Procedural Terminology codes. Patients with a postoperative diagnosis of proximal humerus fracture (International Classification of Diseases-9 codes 812.0*, 812.00, 812.01, 812.02, 812.03, 812.09, 812.1, 812.10, 812.11, 812.12, 812.13, 812.19, 812.20; ICD-10 codes S42.2*, S42.20, S42.201, S42.202, S42.209, S42.21, S42.22, S42.23, S42.24, S42.25, S42.26, S42.29, S42.292, and S42,293) were then selected. Patients who had disseminated cancer; were paralyzed, mentally altered, and unresponsive for more than 24 hours; or had an infection at the time of surgery were excluded because these factors are known risk factors of postoperative/perioperative complications. An assumption was made that any patient listed as undergoing a total shoulder arthroplasty had a reverse total shoulder arthroplasty conducted because it is not standard to conduct an anatomic total shoulder arthroplasty for the treatment of proximal humerus fractures.

### Variables and Outcome Measures

Patient demographic factors including age, sex, smoking status (those who smoked cigarettes or used other tobacco products in the year before admission), race, ethnicity, body mass index (BMI), and preoperative functional status were included. The latter variable focuses on the patient's ability to complete activities of daily living, including bathing, feeding, dressing, and mobility, during the 30 days before surgery. Patients who were defined as “partially dependent” and “totally dependent,” patients who required either some or total assistance to complete activities of daily living, respectively, were combined into one group designated as “dependent” (Table 1). In addition, data for medical comorbidities, including steroid use, diabetes mellitus, hypertension, chronic obstructive pulmonary disease, dialysis, dyspnea, bleed disorders, and need for preoperative transfusion, were collected.

**Table 1:** Demographics and Clinical Factors in Patients Undergoing Hemiarthroplasty or Total Shoulder Arthroplasty for Proximal Humerus Fracture

Parameter^a^	Overall	Hemiarthroplasty	TSA	*P* ^b^
	N = 1,563	N = 436	N = 1,127	
	Value (SD/%)	Value (SD)	Value (SD)	
Patient factors				
Age, mean, yr	71.6 (9.8)	68.8 (10.9)	72.6 (9.1)	**<0.001**
Male Sex, N (%)	272 (17.4)	93 (21.3)	179 (15.9)	0.025
Smoking, N (%)	215 (13.8)	61 (14)	154 (13.7)	0.894
Race, N (%)				**<0.001**
White	1,015 (84.9)	283 (76.5)	732 (88.6)	
Ethnicity, N (%)				0.265
Hispanic	287 (18.4)	105 (24.1)	182 (16.1)	
BMI, mean, kg/m^2^	31.3 (7.8)	31.4 (8.3)	31.2 (7.7)	0.773
Preoperative functional status^c^, N (%)				
Independent	1,116 (71.4)	345 (79.1)	771 (68.4)	**<0.001**
Totally/partially dependent	73 (4.7)	19 (4.4)	54 (4.8)	
Laboratory values				
Serum sodium, mEq/L	137.7 (3.7)	137.4 (3.6)	137.7 (3.8)	0.058
BUN, mg/dL	18.5 (9.9)	18.2 (9.7)	18.6 (10)	0.254
Serum creatinine, mg/dL	0.9 (0.6)	0.9 (0.6)	0.9 (0.6)	0.893
eGFR, mL/min	73.7 (21.9)	76.8 (22.9)	72.6 (21.4)	**0.001**
Serum albumin, g/dL	3.69 (0.5)	3.69 (0.6)	3.69 (0.5)	0.766
WBC, per 10,000 cells/mcL	8.8 (2.8)	9.2 (3.2)	8.6 (2.6)	0.075
HCT, %	36.0 (5.0)	36.2 (4.9)	36.0 (5.0)	0.614
Platelets, per 1,000/mcL	262.0 (93.2)	261.9 (99.3)	262.0 (90.7)	0.765

BMI = body mass index, BUN = blood urea nitrogen, eGFR = estimated glomerular filtration rate, HCT = hematocrit, TSA = total shoulder arthroplasty, WBC = white blood cell count

a Mean and SD presented for continuous variables. Frequency and percentage presented for categorical variables.

b *P* values determined using the chi square test of homogeneity or Fisher exact test for categorical variables and the Mann-Whitney *U* test or Student *t*-test for continuous variables. *P* values have been adjusted for multiple comparisons through the Benjamini-Hochberg false discovery rate *P* value adjustment. Significant values are in bold.

c Based on the degree to which a patient can demonstrate the ability to complete activities of daily living including but not limited to bathing, feeding, dressing, toileting, and mobility. The best functional status demonstrated by the patient within 30 days before surgery is reported.

Diabetes mellitus status is reported in the ACS-NSQIP database by treatment with “insulin, non-insulin” agents and “none.” The latter includes both patients without a diagnosis of diabetes and those who have diabetes but are controlled by diet alone. “Non-insulin” indicates a diagnosis of diabetes mellitus with control through oral or other non-insulin agents. Glycated hemoglobin (Hb_A1c_) is not recorded in the ACS-NSQIP database. Consequently, glycemic control was not able to be determined. However, patients with the designation of “none” were considered as not having diabetes mellitus in this study. In addition to demographic factors and comorbidities, clinical variables included preoperative laboratory values such as serum albumin, liver enzymes, blood urea nitrogen, creatinine, partial thromboplastin time, white blood cell count, hematocrit, and platelets. Furthermore, intraoperative variables, such as duration of surgery, American Society of Anesthesiologists (ASA) classification, clinical setting, procedure type, and anesthesia method, were also included (Table 2). The primary outcomes of interest in this study were complications, readmission, thromboembolic events, need for blood transfusion, mortality, and need for revision surgery.

**Table 2:** Intraoperative Factors, Comorbidities, and Outcomes of Patients Undergoing Hemiarthroplasty or Total Shoulder Arthroplasty for Proximal Humerus Fracture

Parameter^a^	Overall	Hemiarthroplasty	TSA	*P* ^b^
	N = 1,563	N = 436	N = 1,127	
	Value (%)	Value (%)	Value (%)	
Intraoperative factors				
Duration of surgery, min (SD)	129.4 (56.3)	135.1 (60.1)	127.2 (54.6)	0.011
Admit to operation, d (SD)	0.8 (3.1)	1.3 (5.3)	0.6 (1.5)	**<0.001**
Length of stay, d (SD)	3.3 (4.7)	4.0 (7.5)	3 (3.0)	0.979
ASA classification				0.704
I-II	481 (30.8)	138 (31.7)	343 (30.4)	
III-IV	1,083 (69.3)	299 (68.6)	784 (69.6)	
Outpatient procedure	139 (8.9)	69 (15.8)	70 (6.2)	**<0.001**
Elective procedure	1,125 (72.0)	280 (64.2)	845 (75.0)	**<0.001**
Comorbidities, N (%)				
Steroid use	65 (4.2)	14 (3.2)	51 (4.5)	0.250
HTN	1,069 (68.4)	289 (66.3)	780 (69.2)	0.276
Diabetes mellitus				**0.032**
None	1,148 (73.4)	307 (70.4)	841 (74.6)	
Insulin	175 (11.2)	64 (14.7)	111 (9.8)	
Non-insulin	240 (15.4)	65 (14.9)	175 (15.5)	
Dialysis	12 (0.8)	7 (1.6)	5 (0.4)	0.051
Preoperative transfusion	23 (1.5)	6 (1.4)	17 (1.5)	0.867
Bleeding disorder	79 (5.1)	17 (3.9)	62 (5.5)	0.243
History of COPD	128 (8.2)	31 (7.1)	97 (8.6)	0.465
Dyspnea^c^	111 (7.1)	26 (6.0)	85 (7.5)	0.333
Outcome				
Thromboembolic events	17 (1.1)	2 (0.5)	15 (1.3)	0.195
Bleeding requiring transfusion	173 (11.1)	49 (11.2)	124 (11.0)	0.919
Surgical site complications	14 (0.8)	7 (1.6)	7 (0.6)	0.092
Unplanned revision surgery	33 (2.1)	8 (1.8)	25 (2.2)	0.684
Unplanned readmission	60 (3.8)	11 (2.5)	49 (4.3)	0.178
Mortality	15 (1.0)	3 (0.7)	12 (1.0)	0.846
All complications	241 (15.4)	64 (14.7)	177 (15.7)	0.636

ASA = American Society of Anesthesiologists, COPD = chronic obstructive pulmonary disease, HTN = hypertension, TSA = total shoulder arthroplasty

a Mean and SD presented for continuous variables. Frequency and percentage presented for categorical variables.

b *P* values determined using the chi square test of homogeneity or Fisher exact test for categorical variables and the Mann-Whitney *U* test or Student *t*-test for continuous variables. *P* values have been adjusted for multiple comparisons through the Benjamini-Hochberg method. Significant values are in bold.

c Dyspnea present with moderate exertion or at rest.

### Statistical Analysis

Demographic and clinical characteristics of the patient sample were described using descriptive statistics using mean and SD for continuous variables and frequency and percentage for categorical variables. Continuous variables were compared using the Kruskal-Wallis test. Categorical variables were compared using the chi square/Fisher tests. Patient variables with *P* value less than 0.2 in the regression were included in a generalized linear model to calculate the adjusted odds ratios (ORs). This selection of patient factors was conducted to minimize potential overadjustment from inputting a large number of variables in the model. Post hoc bivariate analyses were conducted with parametric or nonparametric tests, such as the Student *t*-test or Mann-Whitney *U* test, respectively, according to appropriate statistical parameters. Intragroup comparisons were analyzed between hemiarthroplasty and reverse TSA groups using the Mann-Whitney *U* and 2 × 2 chi square/Fisher tests. A separate multiple logistic regression analysis with penalized maximum likelihood estimation and Firth bias correction was used to identify independent risk factors of unplanned revision surgery, unplanned readmission, total systemic complications, and total local complications. Adjusted ORs along with 95% confidence interval (CI) were reported. An estimated OR of > 1 indicated greater odds of an unplanned revision surgery/readmission and total complications. Statistical analyses were conducted using SAS software, version 9.4 (SAS Institute). The level of significance was set at α = 0.05 (two-tailed).

## Results

A total of 1,563 patients underwent surgery for the treatment of proximal humerus fracture. The median age of the cohort was 71.6 ± 9.8 years; 17.4% were male (272 patients); 18.4% were Hispanic; and 84.9% were White. Smoking in the year before surgery was present in patients (13.8%), and most patients were functionally independent (71.4%). The median BMI was 31.3 ± 7.8. Four hundred thirty-six patients (27.9%) underwent hemiarthroplasty, and 1,127 patients (71.1%) underwent reverse total shoulder arthroplasty for fracture treatment. Patient demographics and clinical characteristics are presented in Table 1. The reverse TSA group was significantly older (*P* < 0.001), but there was no significant difference between groups for sex, smoking status, or BMI.

The mean surgical time was 129.4 ± 56.3 minutes. The average length of hospital stay was 3.3 ± 4.7 days. Most of the surgeries (72.0%) were conducted as elective procedures (64.2% hemiarthroplasty group; 75.0% reverse TSA group), and these differences were statistically significant (*P* < 0.001). Most of the patients (69.3%) had an ASA classification of >3. 8.9% of the surgeries were conducted as outpatient procedures (15.8% hemiarthroplasty group; 62% reverse TSA group), and these differences were statistically significant (*P* < 0.001). No difference was observed between those who underwent hemiarthroplasty or reverse TSA in duration of surgery, ASA classification, medical comorbidities, or chronic steroid use (Table 2).

**Figure 1:**
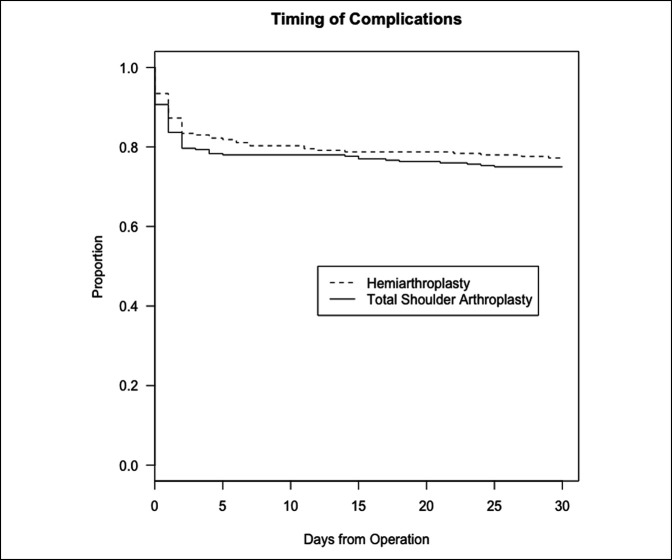
A Kaplan-Meier curve represented as a proportion showing the timing of postoperative complication; the dotted line represents the hemiarthroplasty group and the solid line represents the reverse TSA group demonstrating no difference between groups.

The overall complication rate was 15.4% (14.7% hemiarthroplasty group; 15.7% reverse TSA group [*P* = 0.64]). The incidence of thromboembolic events was 1.1% (0.5% hemiarthroplasty; 1.3% reverse TSA). 11.1% of patients required postoperative blood transfusion for acute blood loss anemia (11.2% hemiarthroplasty; 11.0% reverse TSA). The incidence of surgical site complications was 0.8% (1.6% hemiarthroplasty; 0.6% reverse TSA). Unplanned revision surgery occurred in 2.1% of patients (1.8% hemiarthroplasty; 2.2% reverse TSA). The unplanned readmission rate was 3.8% (2.5% hemiarthroplasty; 4.3 reverse TSA). The overall mortality rate was 1.0% (0.7% hemiarthroplasty; 1.0% reverse TSA). No significant difference was observed in postoperative complications between those who underwent hemiarthroplasty or reverse TSA in this cohort (Figure 1). A Kaplan-Meier curve represented as a proportion shows the timing of postoperative complication by a dotted line representing the hemiarthroplasty group and a solid line representing the reverse TSA group demonstrating no difference between the groups.

Logistic multivariate regression analysis for 30-day complication results are summarized in Tables 3–6 and are broken down by procedure (Table 3 hemiarthroplasty or reverse TSA, Table 4 hemiarthroplasty, and Table 5 reverse TSA). Age older than 65 years (OR, 1.78; 95% CI, 1.23–2.61), male sex (OR, 1.59; 95% CI, 1.12–2.24), preoperative transfusion (OR, 3.62; 95% CI, 1.37–9.10), hypoalbuminemia (OR, 2.11; 95% CI, 1.41–3.15), ASA >3 (OR, 2.01; 95% CI, 1.42–2.89), dialysis (OR, 5.59; 95% CI, 1.48–21.1), thrombocytopenia (OR, 2.75; 95% CI, 1.04–6.78 only for the hemiarthroplasty group), and preoperative sepsis (OR, 3.39; 95% CI, 1.19–8.99 only for the hemiarthroplasty group) were all associated risk factors of complications. Poor kidney function as indicated by estimated glomerular filtration rate <50 mL/min (OR, 2.46; 95% CI, 1.68–3.58), blood urea nitrogen >25 mg/dL (OR, 1.89; 95% CI, 1.31–2.70), serum creatinine >1.2 mg/dL (OR, 2.39; 95% CI, 1.61–3.51), anemia (OR, 2.87; 95% CI, 2.11–3.92), impaired coagulation (OR, 2.20; 95% CI, 1.27–3.74), duration of surgery >106 minutes (OR, 1.46; 95% CI, 1.07–2.01), and length of stay >2.5 days (OR, 2.55; 95% CI 1.81–3.63) all were predictive of an increased risk of postoperative complication. Adjusted analysis focusing only on the hemiarthroplasty group (Table 4) found that poor kidney function (glomerular filtration rate <60 mL/min) (OR, 1.97; 95% CI, 1.97–2.85), anemia (OR, 2.71; 95% CI, 1.84–4.06), and impaired coagulation (OR, 2.23; 95% CI, 1.20–4.02) were factors associated with increased risk of complications. The analysis focusing only on the reverse TSA group (Table 5) found that those with anemia (OR, 2.67; 95% CI, 1.37–5.41), impaired coagulation (OR, 5.15; 95% CI, 1.74–15.5), and length of stay >2.5 days (OR, 2.37; 95% CI, 1.27–4.53) were at increased risk of postoperative complications. BMI >36 kg/m^2^ was found to be protective and decreased the risk of postoperative complications.

**Table 3:** Adjusted Odds Ratios for Complications in the 30-Day Perioperative Period for Proximal Humerus Fractures Treated With Hemiarthroplasty or Reverse Total Shoulder Arthroplasty

Parameter^a^	OR^a^	95% CI
Male sex	1.45	0.97–2.13
BMI >25.0 kg/m^2^	0.82	0.57–1.20
eGFR <50 mL/min	**2.46**	**1.68–3.58**
Anemia	**2.20**	**1.55–3.17**
Thrombocytopenia	1.24	0.71–2.12
Dialysis	2.24	0.57–9.57
Impaired coagulation	**2.20**	**1.27–3.74**
Dyspnea	1.20	0.65–2.09
Duration of surgery >106 min	**1.46**	**1.04–2.06**
Days admit to surgery >2 d	1.29	0.81–2.02
ASA class III-IV	1.36	0.93–2.04
Total shoulder arthroplasty	1.01	0.71–1.45
Total LOS >2.5 d	**2.55**	**1.81–3.63**

95% CI = 95% confidence interval, ASA = American Society of Anesthesiologists, BMI = body mass index, eGFR = estimated glomerular filtration rate, LOS = length of stay, OR = odds ratio

a Significant ratios are in bold.

**Table 4:** Adjusted Odds Ratios for Complications in the 30-Day Perioperative Period for Proximal Humerus Fractures Treated With Hemiarthroplasty

Parameter^a^	OR^a^	95% CI
Age >65 yr	1.01	0.63–1.67
BMI >35 kg/m^2^	0.75	0.48–1.13
eGFR <60 mL/min	**1.97**	**1.36–2.85**
Anemia	**2.71**	**1.84–4.06**
Thrombocytopenia	1.06	0.53–1.99
Steroid use	0.65	0.22–1.57
Impaired coagulation	**2.23**	**1.20–4.02**
Outpatient setting	0.66	0.25–1.50
Elective surgery	0.85	0.58–1.26

95% CI = 95% confidence interval, BMI = body mass index, eGFR = estimated glomerular filtration rate, OR = odds ratio

a Significant ratios are in bold.

**Table 5:** Adjusted Odds Ratios for Complications in the 30-Day Perioperative Period for Proximal Humerus Fractures Treated With Reverse Total Shoulder Arthroplasty

Parameter^a^	OR^a^	95% CI
Male sex	0.80	0.34–1.73
eGFR <50 mL/min	2.17	0.99–4.56
Anemia	**2.67**	**1.37–5.41**
Dialysis	1.98	0.26–18.2
Impaired coagulation	**5.15**	**1.74–15.5**
Dyspnea	1.09	0.29–3.30
Duration of surgery >97 min	1.51	0.74–3.32
Total LOS >2.5 d	**2.37**	**1.27–4.53**

95% CI = 95% confidence interval, OR = odds ratio

a Significant ratios are in bold.

**Table 6:** Crude Odds Ratios of Patients Undergoing Hemiarthroplasty or Total Shoulder Arthroplasty for All Complications

Parameter	Overall		Hemiarthroplasty		TSA	
	N = 1,563		N = 436	N = 1,127	
	OR^a^	95% CI	OR^a^	95% CI	OR^a^	95% CI
Patient factors						
Age >65 yr	**1.78**	**1.23–2.61**	**2.08**	**1.10–4.15**	**1.63**	**1.03–2.65**
Male sex	**1.59**	**1.12–2.24**	0.83	0.38–1.67	**2.07**	**1.38–3.09**
Smoking	0.91	0.59–1.38	0.86	0.33–1.95	0.93	0.55–1.52
White race	1.14	0.77–1.75	1.16	0.60–2.37	1.11	0.66–1.95
Hispanic	1.31	0.73–2.24	1.39	0.33–4.50	1.28	0.66–2.35
BMI >36 kg/m^2^	**0.57**	**0.38–0.85**	**0.36**	**0.12–0.88**	0.66	0.41–1.01
Dependent functional status	0.93	0.67–1.27	0.63	0.26–1.35	1.01	0.70–1.44
Preoperative transfusion	**3.62**	**1.37–9.10**	6.01	0.79–45.9	2.99	0.90–8.96
Laboratory values						
BUN >25 mg/dL	**1.89**	**1.31–2.70**	1.57	0.70–3.31	**2.00**	**1.31–3.03**
Serum creatinine >1.2 mg/dL	**2.39**	**1.61–3.51**	**2.99**	**1.36–6.34**	**2.20**	**1.37–3.47**
Hypoalbuminemia	**2.11**	**1.41–3.15**	1.62	0.76–3.42	**2.34**	**1.43–3.81**
WBC >11,000 cells/mm^3^	0.93	0.62–1.36	1.05	0.52–2.04	0.87	0.52–1.41
Anemia	**2.87**	**2.11–3.92**	**3.66**	**1.95–7.20**	**2.63**	**1.85–3.79**
Thrombocytopenia	1.62	0.94–2.71	**2.75**	**1.04–6.78**	1.26	0.62–2.4
Intraoperative factors						
Duration of surgery >106 min	**1.46**	**1.07–2.01**	1.61	0.85–3.22	1.43	1.00–2.06
ASA classification III-IV	**2.01**	**1.42–2.89**	**1.97**	**1.01–4.10**	**2.03**	**1.35–3.11**
Outpatient setting	1.69	0.95–3.24	2.45	0.94–8.13	1.28	0.62–2.99
Elective procedure	0.55	0.41–0.74	0.34	0.19–0.61	0.65	0.45–0.94
Comorbidities, N (%)						
Steroid treatment	0.88	0.38–1.82	2.41	0.53–8.69	0.57	0.17–1.46
HTN	1.21	0.89–1.66	1.14	0.63–2.13	1.23	0.85–1.81
Diabetes mellitus	1.27	0.93–1.74	1.30	0.70–2.34	1.27	0.87–1.84
Insulin vs none	1.36	0.87–2.09	1.45	0.65–3.05	1.34	0.76–2.26
Non-insulin vs none	1.21	0.81–1.78	1.15	0.48–2.50	1.23	0.77–1.92
Dialysis	**5.59**	**1.48–21.1**	4.50	0.64–27.3	8.15	0.93–98.2
Bleeding disorder	**3.48**	**2.07–5.75**	**7.39**	**2.42–23.0**	**2.75**	**1.49–4.94**
History of COPD	1.02	0.59–1.69	1.13	0.33–3.15	0.98	0.51–1.77
Dyspnea	1.22	0.70–2.05	1.06	0.26–3.28	1.27	0.67–2.28
Preoperative sepsis/SIRS	1.88	0.96–3.51	**3.39**	**1.19–8.99**	1.26	0.46–3.00

95% CI = 95% confidence interval, ASA = American Society of Anesthesiologists, BMI = body mass index, BUN = blood urea nitrogen, COPD = chronic obstructive pulmonary disease, HTN = hypertension, SIRS = systemic inflammatory response syndrome, TSA = total shoulder arthroplasty, WBC = white blood cell count

a Significant ratios are in bold.

## Discussion

This study comparing the 30-day complication rates for patients treated with either hemiarthroplasty or reverse TSA was 15.4%. In addition, no notable difference was found in the incidence of complications, readmissions, revision surgery, surgical site complication, mortality, thromboembolic events, or need for postoperative blood transfusion between groups in the first 30 days after surgery. The management of proximal humeral fractures can be challenging, with outcomes based on the facture pattern, preoperative patient factors, and nonsurgical versus surgical decision making. HA has traditionally been used for proximal humerus fractures in active patients, aged 40 to 65 years, with complex three-part to four-part fractures or head split patterns, when open reduction/internal plate fixation is not feasible.^1^ Reverse TSA is growing as an alternative to hemiarthroplasty with comparable clinical outcomes in the appropriate surgical candidate. On comparison of 30-day postoperative complications and patient-associated risk factors between the two surgical options, more conclusions may be drawn on predicting outcomes in the early postoperative period after surgical management for displaced proximal humerus fractures.

Given the increasing prevalence of total shoulder arthroplasty in elderly patients, especially in those with osteoporotic bone where tuberosity healing is critical and difficult to achieve with hemiarthroplasty, our analysis demonstrates similar patient risk factors and 30-day complication rates with reverse TSA when compared with hemiarthroplasty. Previous studies comparing reverse TSA with hemiarthroplasty with short-intermediate−term follow-up have yielded mixed results for functional outcomes, revision rates, and complications.^5,10,12,16,17,18,19,20^ Bonnevialle et al^21^ conducted a multicenter retrospective study finding higher adjusted constant scores and increased forward flexion, with lower revision rates in the reverse TSA group. van der Merwe et al^22^ reported the functional outcomes and revision rates and found no notable difference in function outcomes/revision at 6 months and 5 years postoperatively. Wang et al^16^ conducted a meta-analysis comparing hemiarthroplasty with reverse TSA and found a 22% overall complication rate in the hemiarthroplasty group and 8.57% complication rate in the reverse TSA group (OR, 2.97; 95% CI, 1.33–6.66). Although more research will be beneficial for predicting long-term outcomes, our study shows similar 30-day complication rates with similar patient risk factors of complication between the two surgical groups. Cuff et al^20^ compared the results of hemiarthroplasty with reverse TSA for complex three-part and four-part proximal humerus fractures. The authors found that the patients who underwent reverse TSA had better patient-reported outcome scores, better tuberosity healing, and better postoperative forward elevation. Thirteen percent of the hemiarthroplasty group elected to undergo revision to reverse TSA because of failed tuberosity healing and pseudoparalysis. No notable difference was observed in complications.

Our data demonstrated a 15.4% mean complication rate with reverse TSA (n = 177) and hemiarthroplasty (n = 64) for the treatment of complex proximal humerus fractures in the early 30-day postoperative period. Patient preoperative comorbidities including past medical conditions (ie, hypertension, diabetes mellitus [DM], dialysis, congestive heart failure [CHF], and chronic obstructive pulmonary disease) showed no statistical difference between the two treatment groups. Surgical factors including duration of surgery, admission before operation, and length of stay were also not statistically different. Hemiarthroplasty was conducted more frequently in the outpatient setting (15.8%) compared with reverse TSA (6.2%), which may factor into a surgeon's experience and comfort level when determining location for hemiarthroplasty versus reverse TSA. Postoperative blood transfusion was reported to have the highest complication rate encountered in this study (reverse TSA n = 124, 11%; hemiarthroplasty n = 49, 11.2%). Given the patient demographics of this patient population, this injury and treatment modality is often conducted on older patients (mean age 71.6 years) with more complex medical comorbidities where there tends to be a higher prevalence of blood transfusion because of patients being less likely to compensate systemically for blood loss and degree of surgical complexity in managing such an injury. Patients experienced thromboembolic events in each group: 1.3% for reverse TSA (n = 15) and 0.5% for hemiarthroplasty (n = 2), with an overall 30-day mortality rate of 11% (17/1,563 patients). This demonstrates the potential for serious complications contributing to morbidity/mortality in this group of patients; our data suggest that this risk is similar between reverse TSA and hemiarthroplasty. Understanding patient demographics, risk factors, and intraoperative requirements can give a better understanding of how to manage these complex injuries and what to expect postoperatively.

Schairer et al^5^ used the National Inpatient Sample database for 2011 to compare hemiarthroplasty with reverse TSA for the treatment of proximal humerus fractures. Similarly, they found no notable differences between groups in postoperative complications, either local or systemic; length of stay; or need for postoperative blood transfusion. Unlike our results, this study demonstrated a higher percentage of patients having hemiarthroplasty (72.6%) and only 27.4% reverse TSA, whereas our data had almost the complete opposite distribution, 27.9% hemiarthroplasty and 71.1% reverse TSA. It is difficult to determine how this may affect the results of either study, but given that both database analyses found that there is no difference in early postoperative complication, the comparison of the study results strengthens the results. The authors reported a 5.3% rate of systemic complications and 2.6% local complication rate, which is lower than our 15.4% overall complication rate in previous series.

Based on 30-day postoperative data, our study examined which patient risk factors may contribute to early complications. The mean age at the time of surgery was 71.6 ± 9.8 years, with the reverse TSA age significantly higher (72.6 ± 9.1 years) as compared with an average of 68.8 ± 10.9 years in the hemiarthroplasty group (*P* < 0.001). Although there was a notable difference between groups, this did not demonstrate to be a patient factor associated with increased risk of complication. Age older than 65 years (OR, 2.08; 95% CI, 1.10–4.15 hemiarthroplasty group; OR, 1.63; 95% CI, 1.03–2.65 reverse TSA group) contributed to the risk of complication. BMI >36 (OR, 0.57; 95% CI, 0.38–0.85) seems to be protective from early risk of postoperative complications. This contradicts the literature in hip/knee arthroplasty where higher BMI leads to increased postoperative complications, including infection and wound healing.^23^ Anemia (OR, 2.20; 95% CI, 1.55–3.17) and impaired coagulation (OR 2.20; 95% CI, 1.27–3.74) were both risks in each group for early complication. Given the higher incidence for anemia, transfusion, and comorbidity in this age group, providers should plan accordingly both preoperatively and postoperatively, regardless of the type of operation. Hypoalbuminemia is often a concern for wound complications and may indicate nutritional deficiency. Although we reported only 14 surgical site complications, hypoalbuminemia is a risk factor of early 30-day complication (OR, 2.11; 95% CI, 1.41–3.15) likely representing overall nutritional status in these patients. Among comorbidities that we identified as a high risk of early complication, high ASA classification (ASA >3) (OR, 2.01; 95% CI, 1.42–2.89) and bleeding disorders (OR, 3.48; 95% CI, 2.07–5.75) corresponded to increased risk of complications in both groups. Finally, we found that hospital length of stay >2.5 days had 2.37× increased risk of early postoperative complications in those patients who underwent reverse TSA (OR, 2.37; 95% CI, 1.27–4.53). Patients who required a prolonged postoperative length of stay may have more medical comorbidities than those who have a shorter length of stay, putting them at increased risk of complications. Elective procedures (OR, 0.55; 95% CI, 0.41–0.74) had a lower OR for complications in the first 30 days, regardless of procedure type versus inpatient. This may be attributed to inpatient surveillance in the immediate postoperative period that leads to a higher identification rate of complications versus outpatient follow-up where complications may go undocumented because of resolution or avoidance of nosocomial-related early complications. In addition, those patients who met criteria to have surgery done as an elective procedure may have had less medical comorbidities than those who required admission at the time of presentation for proximal humerus fracture. Unfortunately, the NSQIP data set does not allow for capturing of these patient-specific details.

The study limitations include 30-day data collection of reported complications. This does not specifically address implant failure, implant longevity, or poor functional outcomes between the groups that often lead to complication and revision surgery outside of the first 30 days. Hemiarthroplasty may require revision to reverse TSA because of tuberosity malunion or nonunion and rotator cuff dysfunction, resulting in a second surgery. Additional studies looking at patient factors leading to these outcomes would help guide management for which patients are best suited for hemiarthroplasty or reverse TSA. This study included 1,563 total patients, undergoing hemiarthroplasty or reverse TSA for the treatment of a proximal humerus fracture, for whom we expect the 30-day complication rate to be relatively low. The sample size in each group may not be large enough to demonstrate a difference between groups, but a study such as this is not ameniabkle to sample size power calculations. A larger study with more patients in each arm would enhance the study's power, potentially leading to additional statistically significant complications. Finally, the NSQIP database was used in this study. Preoperative characteristics, intraoperative data, and postoperative events are recorded from the electronic medical records based on the interpretation of the providers' notes and surgical reports. This may lead to a deficiency with reporting accuracy within the database.

The management of proximal humerus fractures is often a complex decision involving surgical and nonsurgical management strategies. The surgical decision between hemiarthroplasty versus reverse total arthroplasty should include preoperative patient factors, types of injuries, differences in long-term functional outcomes, and overall financial costs of care to determine the best surgical option for the patient. Longer follow-up and additional comparison studies between the two options will help in the creation of proximal humerus fracture treatment guidelines. Our study demonstrates that 30-day complication rates are similar between hemiarthroplasty and reverse TSA. Surgeons should consider all risk factors inherent to both surgical groups to better predict complications and direct management.
